# Incentivised chronic disease management and the inverse equity hypothesis: findings from a longitudinal analysis of Scottish primary care practice-level data

**DOI:** 10.1186/s12916-017-0833-5

**Published:** 2017-04-11

**Authors:** Richard Lowrie, Alex McConnachie, Andrea E. Williamson, Evangelos Kontopantelis, Marie Forrest, Norman Lannigan, Stewart W. Mercer, Frances S. Mair

**Affiliations:** 1 Pharmacy and Prescribing Support Unit, NHS Greater Glasgow and Clyde, Glasgow, Scotland G3 8SJ UK; 2 Robertson Centre for Biostatistics, Institute of Health and Wellbeing, University of Glasgow, Glasgow, Scotland UK; 3 General Practice and Primary Care, School of Medicine, MVLS, University of Glasgow, Glasgow, Scotland UK; 4 The Farr Institute of Health Informatics Research, University of Manchester, Manchester, England UK; 5 East Glasgow Health and Social Care Partnership, Paradise Health Centre, Glasgow, Scotland UK; 6 Institute of Health and Wellbeing, University of Glasgow, Glasgow, Scotland UK

**Keywords:** Primary health care, General practice, Pay-for-performance, Socioeconomic factors, Disparities

## Abstract

**Background:**

The inverse equity hypothesis asserts that new health policies initially widen inequality, then attenuate inequalities over time. Since 2004, the UK’s pay-for-performance scheme for chronic disease management (CDM) in primary care general practices (the Quality and Outcomes Framework) has permitted practices to except (exclude) patients from attending annual CDM reviews, without financial penalty. Informed dissent (ID) is one component of exception rates, applied to patients who have not attended due to refusal or non-response to invitations. ‘Population achievement’ describes the proportion receiving care, in relation to those eligible to receive it, including excepted patients. Examination of exception reporting (including ID) and population achievement enables the equity impact of the UK pay-for-performance contract to be assessed. We conducted a longitudinal analysis of practice-level rates and of predictors of ID, overall exceptions and population achievement for CDM to examine whether the inverse equity hypothesis holds true.

**Methods:**

We carried out a retrospective, longitudinal study using routine primary care data, analysed by multilevel logistic regression. Data were extracted from 793 practices (83% of Scottish general practices) serving 4.4 million patients across Scotland from 2010/2011 to 2012/2013, for 29 CDM indicators covering 11 incentivised diseases. This provided 68,991 observations, representing a total of 15 million opportunities for exception reporting.

**Results:**

Across all observations, the median overall exception reporting rate was 7.0% (7.04% in 2010–2011; 7.02% in 2011–2012 and 6.92% in 2012–2013). The median non-attendance rate due to ID was 0.9% (0.76% in 2010–2011; 0.88% in 2011–2012 and 0.96% in 2012–2013). Median population achievement was 83.5% (83.51% in 2010–2011; 83.41% in 2011–2012 and 83.63% in 2012–2013). The odds of ID reporting in 2012/2013 were 16.0% greater than in 2010/2011 (*p* < 0.001). Practices in Scotland’s most deprived communities were twice as likely to report non-attendance due to ID (odds ratio 2.10, 95% confidence interval 1.83–2.40, *p* < 0.001) compared with those in the least deprived; rural practices reported lower levels of non-attendance due to ID. These predictors were also independently associated with overall exceptions. Rates of population achievement did not change over time, with higher levels (higher remuneration) associated with increased rates of overall and ID exception and more affluent practices.

**Conclusions:**

Non-attendance for CDM due to ID has risen over time, and higher rates are seen in patients from practices located in disadvantaged areas. This suggests that CDM incentivisation does not conform to the inverse equity hypothesis, because inequalities are widening over time with lower uptake of anticipatory care health checks and CDM reviews noted among those most in need. Incentivised CDM needs to include incentives for engaging with the ‘hard to reach’ if inequalities in healthcare delivery are to be tackled.

## Background

Pay-for-performance systems exist in an increasing number of countries [1–6] as a means of improving the quality of healthcare [7–9]. Where incentivised chronic disease management (CDM) has been implemented, small improvements in quality have been reported [10, 11]. However, physicians, incentivised to reach CDM targets, run the risk of providing inappropriate care to achieve performance targets [12], or of excepting an unlimited number of patients within a system in which external inspections are not comprehensive and of unknown effectiveness [2] or widening inequalities through deselection of patients who are more difficult to reach, or for whom incentivised targets are more difficult to achieve [11, 13, 14]. Inequalities in health outcomes, service experiences and variations in care led UK primary care services to introduce the pay-for-performance contract (Quality and Outcomes Framework (QOF)) in 2004 [15, 16]. The CDM contract ended in Scotland in April 2016 but continues in the rest of the UK. It financially incentivises CDM in general practices in the UK, through promoting creation of chronic disease registers and implementation of evidence-based care (as measured by a range of quality indicators) for CDM [17]. Unlike other incentivised CDM schemes [18–20], UK physicians can remove (except) ‘unsuitable’ patients from the denominator for any of the reasons given in Box 1, but because these reasons are seen as being beyond their reasonable control, excepted patients are not included in the calculation of a practice’s achievement against that indicator. Practice funding is reduced pro rata for exception reporting, and maximum payment is calculated through a system of achievement thresholds corresponding to points and payments for each indicator [21, 22]. Exception reporting was included in the UK contract as part of a high-trust system to ensure that practices providing a quality service do not lose out on payments through factors outside their control (Box 1) [22]. Overall exceptions are the sum total of the exceptions given in Box 1.

Exception categories 1–3 act as a safeguard against over or inappropriate intervention, or prevent practices being penalised for factors outwith their control. Category 4, ‘informed dissent’ (ID), differs because practices assign it to patients who could benefit from receiving appropriate care, but for whom the practice and patient have not managed to arrange a consultation. The incentive scheme is UK wide, but there is a difference between England and Scotland in the way ID is reported: the Scottish reporting system captures this reason under ID (Box 1) but English reporting systems include exceptions due to non-attendance after three invitations under the category ‘unsuitable’ [23]. The most recent publication describing practice-level ID rates in England was based on a subset of 37 indicators in 2008/9 [23]. The rate was 0.44%, accounting for 30.1% of exceptions overall. The criteria constituting ID in Scotland enables an examination of rates and predictors of non-engagement among all patients who are eligible to receive incentivised activities.

Exception rates quantify failed opportunities for practices and patients to engage in preventative CDM (an ‘engagement gap’) and for patients to receive appropriate care. Exception reporting is under practice control and may serve practices’ financial self-interests [24, 36].

After removing excepted patients from the denominator, practices can receive the maximum payment by achieving targets for a proportion of the remaining patients (‘reported achievement’) [17]. This highlights the scheme’s potential for a practice to achieve maximum points without covering the practice’s total population, thereby risking low uptake of preventative evidence-based interventions among hard to reach groups who may be most in need [25]. By omitting excepted patients, the reported achievement may also mask wider inequalities [26], and examining CDM performance reporting data without taking account of exceptions runs the risk of underestimating the extent of inequalities [27]. ‘Population achievement’ (Box 2) is a preferred measure of practice-level achievement because numbers achieving the indicator are expressed as a proportion of all patients, including those who are excepted [24, 28].

Exception reporting has been described as a quality marker because high rates can suggest that patient preferences are being considered [29]; however, qualitative [30, 31] and quantitative [17, 23] reports indicate that some practices use exception reporting at the end of the contract year, to help reach payment targets.

Patient-level exception reporting data have suggested that non-attendance accounts for one third of ID, and expressed dissent for two thirds of ID [32]. ID and overall exception rates are increasing over time, with predictors including increasing age and extent of deprivation [33].

Systematic reviews provide weak evidence of financial incentives reducing socioeconomic inequalities in CDM [32, 35] and health inequalities are believed to have diminished over time after the introduction of the pay-for-performance scheme [11, 34, 36]. During the initial years of the scheme, analyses suggested that the inverse equity hypothesis (which suggests that policy initiatives for health interventions initially benefit people of higher socioeconomic status and only later benefit people of lower socioeconomic status) [37] did apply [11, 38, 39]; however, we are not aware of any study examining practice-level exception data from pay-for-performance in the UK after 2009. Exception rates and population achievement over time are recommended as more appropriate measures to evaluate the impact of the programme on health equity [26, 40]. The absence of longitudinal analyses of practice-level ID, overall exception reporting rates and population achievement in the same practices using the same indicators has limited our understanding of the equity impact of the incentivised UK CDM programme.

In this study, we used longitudinal practice-level data to investigate rates, predictors and trends over time of ID exception reporting, overall exception reporting and population achievement in order to determine whether the inverse equity hypothesis holds true for the incentivised UK CDM programme.

## Box 1: Reasons for exception reporting as recorded in Scotland^a^

1. Unsuitable (U)

• Patients for whom it is not appropriate to review the chronic disease parameters due to particular circumstances, for example, terminal illness, extreme frailty.

• Patients who are on maximum tolerated doses of medication whose levels remain suboptimal.

• Patients for whom prescribing a medication is not clinically appropriate, for example, those who have an allergy.

• Where a patient has not tolerated medication.

• Where the patient has a supervening condition which makes treatment of their condition inappropriate, for example, cholesterol reduction in liver disease.

2. Registration date and diagnosis date (R)

• Patients newly diagnosed within the practice or who have recently registered with the practice, who should have measurements made within 3 months and delivery of clinical standards within 9 months.

3. Other (O)

• Where an investigative service or secondary care service is unavailable.

4. Informed dissent (ID)

• Patients who have been recorded as refusing to attend review who have been invited on at least three occasions during the preceding 12 months.

• Where a patient does not agree to investigation or treatment, and this has been recorded in their medical records.

^a^ If more than one exception reporting criteria are attached to the patient record, the patient is counted as having been exception reported for the first reason. Patients with multiple conditions covered by QOF who do not respond to invitations will be counted multiple times in that year. Practices may include multiple exception reasons but the Scottish QOF reporting system captures only the first reason listed.

## Box 2: Formulae used to calculate rates for each indicator

$$ \mathrm{Rate}\ \mathrm{o}\mathrm{f}\ \mathrm{n}\mathrm{o}\mathrm{n}\ \mathrm{attendance}\ \mathrm{due}\ \mathrm{t}\mathrm{o}\ \mathrm{ID} = \kern0.5em \frac{\mathrm{ID}\ {\mathrm{exceptions}}^a \times 100}{{\mathrm{Indicator}\ \mathrm{denominator}}^b} $$

$$ \mathrm{Total}\ \left(\mathrm{Overall}\right)\ \mathrm{exception}\ \mathrm{rate} = \kern0.5em \frac{\mathrm{All}\ {\mathrm{exceptions}}^{a\ }\kern0.5em  \times 100}{{\mathrm{Indicator}\ \mathrm{denominator}}^b} $$

$$ \mathrm{Population}\ \mathrm{achievement} = \kern0.5em \frac{\mathrm{Number}\ \mathrm{of}\ \mathrm{cases}\ \mathrm{achieving}\ \mathrm{indicator}\ \mathrm{target} \times 100}{{\mathrm{Indicator}\ \mathrm{denominator}}^b} $$

^a^ Number of patients with each pre-specified disease in each general practice, who have an entry in their medical records, of ID as the first exception reason

^b^ Number of patients in each general practice, with each pre-specified disease, including those excepted

## Methods

### Data

In Scottish practices, data from the incentivised CDM system (QOF) are automatically extracted from practices’ clinical computing systems and collated by the Information Services Division (ISD; part of National Health Services (NHS) National Services Scotland). Data were obtained in December 2014 after any adjustments agreed between practices and ISD, made to ensure patients who were exception reported had not subsequently been included as having met targets. ID, overall exceptions and population achievement were calculated as shown in Box 2. Patients with multiple morbidities are eligible for multiple indicators, and if they are excepted for one indicator, it is possible that they will be excepted for other indicators. For example, the indicators cholesterol and blood pressure control are relevant across many disease domains and, hence, exception reporting on these measures for a multi-morbid patient will likely be reflected across all of his/her relevant conditions. Another example relevant to numerous conditions was influenza immunisation, with patients most likely excepted due to refusal [33]. If two or more exception codes are associated with a patient, the first entered code is taken, in accordance with business rules for QOF reporting [41]. Some practices may have more patients of this type than others, so the analysis included random effects for practices to allow for clustering of exception reporting at the practice level. ID [23] and overall [17] exception reporting rates vary between practices and clinical indicators, so we included the same indicators from the same practices. To improve comparability of data across 3 years of study, we restricted our analysis to those indicators retaining the same definition for all 3 years (Table 1).

**Table 1:** Included indicators

Indicator	Definition
BP 04	Record of BP
BP 05	Last BP ≤ 150/90 mmHg
COPD 08	Influenza vaccine in the preceding 7 months
COPD 10	Record of forced expiratory volume in 1 s
COPD 13	Review, including MRC (Medical Research Council)
DM 02	Record of body mass index
DM 10	Neuropathy testing
DM 13	Micro-albuminuria testing
DM 17	Last measured total cholesterol ≤ 5 mmol/L
DM 18	Influenza vaccine
DM 21	Retinal screening
DM 22	Record of estimated glomerular filtration rate or creatinine
STROKE 06	TIA or stroke with BP ≤ 150/90 mmHg
STROKE 07	TIA or stroke with a record of total cholesterol
STROKE 08	TIA or stroke with last measured total cholesterol ≤ 5 mmol/L
STROKE 10	TIA or stroke with influenza vaccine
EPILEP 08	On drug treatment and seizure free
EPILEP 06	On drug treatment with a record of seizure frequency
CHD 06	Last BP ≤ 150/90 mmHg
CHD 08	Last total cholesterol ≤ 5 mmol/L
CHD 12	Influenza vaccine
CKD 02	Record of BP
CKD 03	Last BP ≤ 140/85 mmHg
CKD 06	Record of urine albumin to creatinine ratio (or protein to creatinine ratio)
DEM 02	Care has been reviewed
DEP 01	DM/CHD with depression screen
PP 01	New hypertensive medication (without CHD, diabetes, stroke and/or TIA) with a cardiovascular risk assessment
PP 02	Hypertensive medication; given lifestyle advice for physical activity, smoking, alcohol and healthy diet
THYROI 02	Hypothyroidism with thyroid function tests

*BP* blood pressure, *CHD* coronary heart disease, *CKD* chronic kidney disease, *COPD* chronic obstructive pulmonary disease, *DEM* dementia, *DEP* depression, *EPILIP* epilepsy, *DM* diabetes mellitus, *PP* primary prevention of heart disease, *THYROI* thyroid disease, *TIA* transient ischaemic attack

We included practices returning data for all these indicators for each year. This enabled an analysis of predictors independent of interference introduced by the indicator, practice or yearly variation.

### Statistical analysis

We conducted a retrospective analysis of ID, overall exception reporting and population achievement from 1 April 2010 to 31 March 2013.

The dataset for analysis included separate observations for each of 29 indicators measured in 793 practices in three separate years (2011, 2012 and 2013), resulting in a total of 68,991 observations (793 practices × 3 years × 29 indicators). This represented a total of 15.0 million opportunities for exception reporting.

Data on the rates of ID exceptions, total exceptions and population achievement were in the form of the number of patients excepted from or achieving the indicator, and the number of patients eligible for the indicator. We fitted a mixed effects logistic regression model for the proportion of patients who met each outcome across all indicators, practices and years to determine significant practice-level predictors. All models included random effects for practices and indicators. Predictors were modelled as categorical variables and, where appropriate, as continuous variables; the results were broadly similar and the categorical results are presented. We looked at all hypothesised predictors individually and together in a best-fitting model found by backwards selection. All statistical analyses were performed with R software (version 3.0.1) using the lme4 package [42]. Results are presented as odds ratios (OR) compared to the reference category with 95% confidence intervals (CI) and an overall *P* value for the differences between the categories. Any individual patient could contribute to several indicators; however, the unit of analysis was the practice-indicator-year combination so a single patient contributed only once to each unit of analysis. Data summaries show the number of exceptions rather than the number of patients excepted. Our analysis combined indicators and therefore we report exceptions rather than individual patients.

We obtained both practice-level and indicator-level predictors to investigate factors associated with ID exception, overall exception and population achievement rates. These factors included the percentage of the practice population living in an area designated as Scotland’s top 15% most socioeconomically deprived; the mean age of the practice population; practice list size; patients per general practitioner (GP); number of GPs; mid-points of GP age bands; degree of rurality of the practice population; the percentage of patients who had not achieved each indicator in the contract year; and whether the achievement threshold for each indicator had increased during the 3-year period (April 2010 to March 2013). For each continuous variable, cut-offs were chosen to give sufficient numbers for analysis in each subgroup, ideally with approximately equal numbers in each subgroup. The characteristics of the practices varied from year to year; Appendix 1 shows the distribution of the predictor variables in 2010/2011. We describe the unweighted proportion of exceptions for each indicator separately and for all clinical domains combined [11, 17, 23]. The mixed effects logistical regression model used the number of exceptions as the numerator and the number of eligible patients as the denominator, so the analysis is appropriately weighted according to the number of patients for each indicator in each practice. We provide the median, which does not take account of weighting. However, fully weighted percentages are available from the corresponding author on request. Results of dependent variables are expressed as a combination of all 29 included indicators.

## Results

We included data from 793 practices that returned exception reporting data for all 29 QOF indicators retaining the same definition throughout the 3 years (April 2010 to March 2013) of the study.

### Three-year median rates of informed dissent, total exception reporting and population achievement

Table 2 shows the clinical indicators categorised by disease, with the median percentage ID rates, overall exception rates and population achievement from 2010 to 2013.

**Table 2:** Informed dissent exceptions, overall exceptions and population achievement rates (median %) for 2010–2013

	ID			Overall exception reporting			Population achievement		
	2010–2011	2011–2012	2012–2013	2010–2011	2011–2012	2012–2013	2010–2011	2011–2012	2012–2013
Indicator									
BP 04 record of BP^a^	0.78	0.88	1.09	1.87	1.79	2.26	91.30	91.21	91.21
BP 05 last BP^a^ ≤ 150/90 mmHg	0.96	1.11	1.42	4.44	4.56	4.97	78.21	78.34	79.64
COPD 08 influenza vaccine in the preceding 7 months	1.85	1.99	2.19	13.76	13.33	12.68	81.51	81.82	82.26
COPD 10 record of forced expiratory volume in 1 s^b^	3.57	3.91	4.42	14.81	15.79	15.97	78.07	76.64	77.00
COPD 13 review, including MRC (Medical Research Council) score^b^	3.42	3.70	4.23	12.11	12.50	12.15	82.10	81.53	81.93
DM 02 record of body mass index^b^	1.48	1.59	1.75	3.62	3.58	3.56	92.20	91.98	91.89
DM 10 neuropathy testing^b^	2.10	2.27	2.52	6.58	6.56	6.81	86.65	86.72	86.10
DM 13 micro-albuminuria testing^b^	2.24	2.47	2.56	5.67	5.53	5.78	87.23	87.12	86.32
DM 17 last measured total cholesterol ≤ 5 mmol/L^b^	2.24	2.40	2.63	10.71	10.75	11.83	75.38	74.51	74.18
DM 18 influenza vaccine in preceding 7 months	2.16	2.17	2.42	16.02	15.41	14.80	78.26	78.66	78.49
DM 21 retinal screening^b^	1.65	1.73	1.92	9.80	9.53	9.62	85.30	85.49	85.71
DM 22 record of estimated glomerular filtration rate or creatinine^b^	1.23	1.21	1.33	2.24	2.21	2.23	95.45	95.52	95.85
STROKE 06 TIA or stroke with BP^b^ ≤ 150/90 mmHg	0.56	0.66	0.71	4.55	4.88	4.76	85.71	85.62	86.11
STROKE 07 TIA or stroke with a record of total cholesterol^b^	0.85	1.02	1.06	4.55	4.65	4.79	88.99	88.71	89.44
STROKE 08 TIA or stroke with last measured total cholesterol^b^ ≤ 5 mmol/L	1.12	1.39	1.49	12.68	13.24	13.43	70.59	70.49	70.77
STROKE10 TIA or stroke with influenza vaccine in preceding 7 months	0.74	1.03	1.04	15.66	14.53	13.79	78.08	78.81	79.42
EPILEP 08 ≥ 18 years old on drug treatment and seizure free for past 12 months	1.16	1.92	2.08	24.00	24.32	25.00	58.73	58.62	57.41
EPILEP 06 ≥ 18 years old on drug treatment with a record of seizure frequency^b^	0.00	0.00	0.00	4.00	4.94	5.00	91.30	90.57	91.09
CHD 06 last BP^b^ ≤ 150/90 mmHg	0.75	0.71	0.64	3.29	3.17	3.33	88.41	87.94	88.60
CHD 08 last total cholesterol^b^ ≤ 5 mmol/L	1.25	1.26	1.11	10.13	10.46	10.99	75.25	73.24	73.49
CHD 12 influenza vaccine in preceding 7 months	0.99	0.95	0.92	12.90	12.13	11.60	82.40	82.82	82.90
CKD 02 record of BP^b^ in those with CKD	0.00	0.00	0.00	0.56	0.63	0.62	97.35	97.18	97.16
CKD 03 last BP^b^ ≤ 140/85 mmHg	0.00	0.00	0.00	6.46	6.31	6.27	70.97	71.59	72.33
CKD 06 record of urine albumin to creatinine ratio (or protein to creatinine ratio)^b^	0.00	0.00	0.20	3.91	3.85	3.76	83.07	83.02	83.08
DEM 02 care has been reviewed^b^ in those with dementia	0.00	0.00	0.00	5.56	5.17	6.25	75.68	76.77	78.33
DEP 01 DM/CHD with depression screen^b^	0.43	0.51	0.40	3.64	3.64	3.95	87.45	86.75	86.62
PP 01 new hypertensive medication (without CHD, diabetes, stroke and/or TIA)^c^ with a cardiovascular risk assessment using approved tool	0.00	0.00	0.00	12.50	12.50	11.11	73.33	72.22	75.56
PP 02 hypertensive medication; given lifestyle advice^b^ for physical activity, smoking, alcohol and healthy diet	0.00	0.00	0.00	6.67	4.76	3.39	79.55	80.49	82.86
THYROI 02 hypothyroidism with thyroid function tests^b^	0.00	0.00	0.00	0.47	0.52	0.59	95.91	95.80	95.45
All indicators combined	0.76	0.88	0.96	7.04	7.02	6.92	83.51	83.41	83.63

Data are presented as the median percentage. ^a^ in the previous 9 months; ^b^ in the previous 15 months; ^c^ in the past year.

*BP* blood pressure, *CHD* coronary heart disease, *CKD* chronic kidney disease, *COPD* chronic obstructive pulmonary disease, *DEM* dementia, *DEP* depression, *EPILIP* epilepsy, *DM* diabetes mellitus, *PP* primary prevention of heart disease, *THYROI* thyroid disease, *TIA* transient ischaemic attack

Eight clinical domains included more than one indicator: blood pressure (BP), chronic obstructive pulmonary disease (COPD), diabetes mellitus (DM), stroke, coronary heart disease (CHD), primary prevention of heart disease 01 (PP 01), epilepsy and chronic kidney disease (CKD).

Over the 3-year period, median rates of ID increased for 18 indicators, remained unchanged for seven (for which all values were 0 across all 3 years), and decreased for four (CHD indicators and depression (DEP), which was dependent on the CHD indicator). ID exception reporting rates trended in the same direction over time for each indicator within six domains: BP, COPD, DM, stroke, epilepsy and CHD. The highest rates (>1%) applied to indicators within the BP, COPD, DM, stroke and CHD domains. Grouped together by disease, the highest median ID rate was noted for patients with COPD followed by DM. Increases in the reported rates of ID between 2010 and 2013 were modest, for example, a 20% increase for the indicator DM 10. The median ID rate across all practice-indicator-year combinations was 0.86% (interquartile range (IQR) 0.0–3.23%).

Overall exception reporting rates increased for 18 of the 29 indicators over the 3 years, with decreases for the remaining 11. The median overall exception reporting rate was 6.98% (IQR 2.63–13.30%) across all indicators, practices and years.

The population achievement rate for the composite of indicators remained stable in each of the 3 years of analysis. The rate was 83.5% (IQR 75.1–90.7%) across all the practice-indicator-year combinations. For those indicators appearing more difficult to achieve, for example, epilepsy and COPD, corresponding rates of ID were either low (epilepsy 06, 0% for all years) or raised (COPD 10, median rate 4.42% in 2012/2013), suggesting that differences in the level of difficulty achieving the indicator were caused by the nature of the indicator or the ability of patients to attend their practice to receive the indicator. Higher rates of median achievement appeared to correspond with higher rates of ID exception reporting.

### Predictors of informed dissent exception reporting

Table 3 illustrates the associations between hypothesised practice-level predictors and ID. The table shows ORs from models for the combination of all 29 indicators, across 793 practices, over 3 years as univariate analyses and also after controlling for confounding variables.

**Table 3:** Independent predictors of 3-year Quality and Outcomes Framework indicators for informed dissent

Predictor		Univariate (odds ratios)		Best fit (odds ratios)	
		Estimated (95% CI), *P* value	Overall *P* value	Estimated (95% CI), *P* value	Overall *P* value
Year					
	2010/2011^*^ 2011/2012 2012/2013	- 1.070 (1.062–1.079), *P* < 0.001 1.155 (1.146–1.165), *P* < 0.001	*P* < 0.001	- 1.073 (1.065–1.082), *P* < 0.001 1.160 (1.151–1.169), *P* < 0.001	*P* < 0.001
Practice population: % in top 15% most deprived					
	None^*^ <5% 5–15% 15–25% 25–50% >50%	- 1.113 (1.066–1.163), *P* < 0.001 1.879 (1.679–2.102), *P* < 0.001 1.978 (1.760–2.222), *P* < 0.001 2.075 (1.831–2.352), *P* < 0.001 2.095 (1.825–2.404), *P* < 0.001	*P* < 0.001	- 1.084 (1.037–1.132), *P* < 0.001 1.704 (1.514–1.918), *P* < 0.001 1.770 (1.563–2.005), *P* < 0.001 2.028 (1.771–2.322), *P* < 0.001 2.221 (1.914–2.577), *P* < 0.001	*P* < 0.001
Practice population: mean age					
	<40^*^ 40–41 ≥42	- 0.803 (0.661–0.975), *P* = 0.027 0.499 (0.409–0.609), *P* < 0.001	*P* < 0.001	removed during backward stepwise regression	
Practice population: list size					
	<5000^*^ 5–10,000 ≥10,000	- 1.865 (1.581–2.198), *P* < 0.001 2.162 (1.628–2.871), *P* < 0.001	*P* < 0.001	- 1.639 (1.384–1.941), *P* < 0.001 1.895 (1.431–2.509), *P* < 0.001	*P* < 0.001
Practice population: patients per GP					
	<1000^*^ 1000–1500 ≥1500	- 1.393 (1.160–1.673), *P* < 0.001 1.637 (1.319–2.032), *P* < 0.001	*P* < 0.001	removed during backward stepwise regression	
Practice GPs: number of GPs					
	1^*^ 2-3 4–6 ≥7	- 0.882 (0.618–1.258), *P* = 0.489 1.260 (0.884–1.795), *P* = 0.202 1.641 (1.144–2.353), *P* = 0.007	*P* < 0.001	removed during backward stepwise regression	
Practice GPs: mean age					
	≤44^*^ 45–49 ≥50	- 0.934 (0.771–1.131), *P* = 0.483 0.863 (0.699–1.066), *P* = 0.171	*P* = 0.392	removed during backward stepwise regression	
Practice: urban/rural					
	Large urban^*^ Other urban Small town Rural	- 0.847 (0.699–1.026), *P* = 0.089 0.806 (0.632–1.029), *P* = 0.084 0.342 (0.277–0.421), *P* < 0.001	*P* < 0.001	- 0.881 (0.728–1.066), *P* = 0.193 1.055 (0.826–1.349), *P* = 0.667 0.690 (0.546–0.872), *P* = 0.002	*P* = 0.006
Indicator: % not achieved*					
	<5%^*^ 5-10% >10%	- 0.914 (0.906–0.923), *P* < 0.001 0.784 (0.774–0.794), *P* < 0.001	*P* < 0.001	- 0.912 (0.903–0.920), *P* < 0.001 0.783 (0.773–0.793), *P* < 0.001	*P* < 0.001
Indicator: threshold increasing					
	No^*^ Yes	- 0.705 (0.381–1.304), *P* = 0.265	*P* = 0.265	removed during backward stepwise regression	

* Reference group for odds ratio

*CI* confidence interval, *GP* general practitioner

Univariate analyses show ID increased over the 3-year period, and followed a socioeconomic gradient with higher median rates in the most deprived practices. ID was higher in practices with younger populations than in practices with older populations. Median rates increased as practice list size increased; practices with more patients per GP had higher median levels. Large urban practices reported higher rates of ID compared with rural practices.

A model-selection procedure identified a subset of five independent predictors associated with ID in the best-fit model: year of reporting, practice-level deprivation, list size, urban/rural practice location, and extent of indicator non-achievement. The odds of ID exception reporting for patients with at least one of the specified 11 diseases in 2012/2013 was greater than in 2010/2011 after controlling for other predictors (OR 1.16, 95% CI 1.15–1.17, *P* < 0.001). The most deprived practices were more likely to report ID compared with least deprived practices (OR 2.22, 95% CI 1.91–2.58, *P* < 0.001). Practices with the largest list sizes were also more likely to report ID (OR 1.89, 95% CI 1.43–2.51, *P* < 0.001). Significant differences were also seen with practice location (odds of ID reporting were 31% lower in rural areas) and when there were higher rates of QOF non-achievement in the same year: the odds of ID reporting by practices were over 20% lower in lower-achieving practices (practices with higher levels of non-achievement). Practices with higher achievement had higher rates of exception reporting.

### Predictors of overall exception reporting

A model-selection procedure identified a subset of independent predictors of overall exceptions (Table 4) that were similar to those found with ID exceptions. These included further evidence that practices achieving higher points had higher levels of exceptions and that more deprived practices, urban practices, higher list sizes and the progress of time were also associated with higher exceptions.

**Table 4:** Independent predictors of 3-year Quality and Outcomes Framework indicators for overall exceptions

Predictor		Total exceptions			
		Univariate (odds ratios)		Best fit (odds ratios)	
		Estimated (95% CI), *P* value	Overall *P* value	Estimated (95% CI), *P* value	Overall *P* value
Year	2010/2011^*^ 2011/2012 2012/2013	- 0.995 (0.991–1.000), *P* = 0.062 1.024 (1.019–1.029), *P <* 0.001	*P <* 0.001	- 0.997 (0.993–1.002), *P* = 0.293 1.028 (1.023–1.033), *P <* 0.001	*P* < 0.001
Practice population: % in top 15% most deprived	None^*^ <5% 5–15% 15–25% 25–50% >50%	- 1.042 (1.019–1.065), *P <* 0.001 1.178 (1.119–1.239), *P <* 0.001 1.268 (1.202–1.338), *P <* 0.001 1.359 (1.282–1.440), *P <* 0.001 1.418 (1.325–1.517), *P <* 0.001	*P <* 0.001	- 1.035 (1.012–1.059), *P* = 0.002 1.128 (1.068–1.192), *P <* 0.001 1.188 (1.119–1.261), *P <* 0.001 1.278 (1.197–1.365), *P <* 0.001 1.325 (1.227–1.429), *P <* 0.001	*P* < 0.001
Practice population: mean age	<40^*^ 40–41 ≥42	- 0.908 (0.843–0.979), *P* = 0.012 0.785 (0.727–0.847), *P <* 0.001	*P <* 0.001	removed during backward stepwise regression	
Practice population: list size	<5000^*^ 5–10,000 ≥10,000	- 1.137 (1.066–1.213), *P <* 0.001 1.121 (1.003–1.254), *P* = 0.045	*P <* 0.001	- 1.091 (1.021–1.165), *P* = 0.010 1.081 (0.970–1.206), *P* = 0.160	*P* = 0.030
Practice population: patients per GP	<1000^*^ 1000–1500 ≥1500	- 1.092 (1.018–1.171), *P* = 0.014 1.186 (1.092–1.288), *P <* 0.001	*P <* 0.001	removed during backward stepwise regression	
Practice GPs: number of GPs	1^*^ 2–3 4–6 ≥7	- 0.921 (0.802–1.057), *P* = 0.240 0.956 (0.833–1.097), *P* = 0.520 1.008 (0.876–1.159), *P* = 0.911	*P* = 0.150	removed during backward stepwise regression	
Practice GPs: mean age	≤44^*^ 45–49 ≥50	- 0.984 (0.915–1.059), *P* = 0.672 1.025 (0.946–1.111), *P* = 0.549	*P* = 0.594	removed during backward stepwise regression	
Practice: urban/rural	Large urban^*^ Other urban Small town Rural	- 0.856 (0.796–0.921), *P <* 0.001 0.870 (0.792–0.955), *P* = 0.003 0.659 (0.609–0.713), *P <* 0.001	*P <* 0.001	- 0.900 (0.835–0.970), *P* = 0.006 0.971 (0.881–1.070), *P* = 0.548 0.799 (0.727–0.878), *P <* 0.001	*P* < 0.001
Indicator: % not achieved	<5%^*^ 5–10% >10%	- 0.923 (0.918–0.928), *P <* 0.001 0.853 (0.846–0.859), *P <* 0.001	*P <* 0.001	- 0.922 (0.917–0.927), *P <* 0.001 0.852 (0.846–0.858), *P <* 0.001	*P* < 0.001
Indicator: threshold increasing	No^*^ Yes	- 1.204 (0.645–2.248), *P* = 0.560	*P* = 0.560	removed during backward stepwise regression	

* Reference group for odds ratio

*CI* confidence interval, *GP* general practitioner

### Predictors of population achievement

Following a model-selection procedure, Table 5 shows that practice-level deprivation was independently associated with low population achievement: the lowest odds of achievement were associated with the most deprived practices (OR 0.84, 95% CI 0.80–0.87, *P* < 0.001).

**Table 5:** Analysis of 3-year Quality and Outcomes Framework indicators for population achievement

Predictor		Univariate (odds ratios)		Best fit (odds ratios)	
		Estimated (95% CI), *P* value	Overall *P* value	Estimated (95% CI), *P* value	Overall *P* value
Year	2010/2011^*^ 2011/2012 2012/2013	- 0.991 (0.988*–*0.995), *P* < 0.001 1.006 (1.003*–*1.010), *P* < 0.001	*P* < 0.001	- 0.998 (0.994*–*1.001), *P* = 0.164 1.012 (1.008*–*1.015), *P* < 0.001	*P* < 0.001
Practice population: % in top 15% most deprived	None^*^ <5% 5–15% 15–25% 25–50% >50%	- 0.968 (0.954*–*0.982), *P* < 0.001 0.894 (0.865*–*0.924), *P* < 0.001 0.894 (0.863*–*0.926), *P* < 0.001 0.832 (0.800*–*0.864), *P* < 0.001 0.854 (0.816*–*0.895), *P* < 0.001	*P* < 0.001	- 0.980 (0.966*–*0.995), *P* = 0.007 0.940 (0.912*–*0.970), *P* < 0.001 0.912 (0.882*–*0.942), *P* < 0.001 0.862 (0.832*–*0.893), *P* < 0.001 0.837 (0.802*–*0.873), *P* < 0.001	*P* < 0.001
Practice population: mean age	<40^*^ 40–41 ≥42	- 1.037 (0.988*–*1.088), *P* = 0.138 1.117 (1.064*–*1.173), *P* < 0.001	*P* < 0.001	removed during backward stepwise regression	
Practice population: list size	<5000^*^ 5–10,000 ≥10,000	- 0.882 (0.847*–*0.918), *P* < 0.001 0.881 (0.822*–*0.945), *P* < 0.001	*P* < 0.001	- 0.913 (0.881*–*0.947), *P* < 0.001 0.918 (0.863*–*0.975), *P* = 0.006	*P* = 0.003
Practice population: patients per GP	<1000^*^ 1000–1500 ≥1500	- 0.951 (0.910*–*0.995), *P* = 0.028 0.920 (0.873*–*0.970), *P* = 0.002	*P* = 0.005	removed during backward stepwise regression	
Practice GPs: number of GPs	1^*^ 2–3 4–6 ≥7	- 0.999 (0.916*–*1.090), *P* = 0.983 0.949 (0.870*–*1.035), *P* = 0.235 0.908 (0.831*–*0.992), *P* = 0.032	*P* = 0.002	removed during backward stepwise regression	
Practice GPs: mean age	≤44^*^ 45–49 ≥50	- 0.980 (0.936*–*1.027), *P* = 0.396 0.985 (0.936*–*1.036), *P* = 0.553	*P* = 0.683	- 0.999 (0.961*–*1.039), *P* = 0.974 0.951 (0.910*–*0.993), *P* = 0.023	*P* = 0.038
Practice: urban/rural	Large urban Other urban Small town Rural	-* 1.045 (0.996*–*1.096), *P* = 0.073 1.048 (0.986*–*1.114), *P* = 0.130 1.210 (1.149*–*1.275), *P* < 0.001	*P* < 0.001	removed during backward stepwise regression	
Indicator: threshold increasing	No^*^ Yes	- 0.617 (0.373*–*1.020), *P* = 0.060	*P* = 0.060	removed during backward stepwise regression	

* Reference group for odds ratio

*CI* confidence interval, *GP* general practitioner

## Discussion

Our longitudinal analysis of practice-level data from 793 Scottish general practices serving 4.4 million patients showed that more deprived practices were at least twice as likely to report ID compared with the least deprived practices (OR 2.22, 95% CI 1.91–2.58, *P* < 0.001) and 32% more likely to report overall exceptions. Of all the predictor variables included in each of the regression analyses, deprivation was the strongest predictor (based on ORs) in every regression model. This result suggests that there is a lower uptake of appropriate clinical care for CDM that aims to prevent further disability and mortality for those most in need of preventative and ongoing health service intervention.

Rates of ID and overall exception reporting increased over time. High achievement (contract points and therefore remuneration) was associated with higher rates of ID and overall exception reporting, re-opening the possibility of gaming, that is, those who use the exception reporting rules more often achieve higher QOF achievement, or suggesting that higher-achieving practices are more adept at identifying and reporting exceptions.

In three recent CDM contract years, the year of reporting, increased deprivation, an urban location and large practice size were predictive of ID, overall exception reporting rates and population achievement. Contrary to previous findings based on the early years of the contract [11], our findings suggest that the inverse equity hypothesis does not hold, because practices in more deprived areas are more likely to show higher rates of exceptions, implying that more patients are missing out on CDM opportunities. Our study raises the possibility that the incentivised CDM programme, recently curtailed in Scotland but continuing to operate in the rest of the UK, may be widening the health equity gap by not directly incentivising practices to use methods of engagement other than letters, for example, house visits or assertive outreach. Given the central importance of primary care, the finding of a lower uptake among patients with at least one long-term condition who are living in more deprived backgrounds suggests the UK contract may not lead to an increase in health status, or not act in such a way as to decrease the higher use of accident and emergency facilities, emergency admissions and out-of-hours clinic use seen in patients from more deprived areas [43]. Practice-level CDM contract payment mechanisms do not take into account patient-level multi-morbidity or an individual’s other circumstances, for example, their ability to travel to their practice for multiple appointments [24, 44, 45]. Our data raise the possibility that the persisting ill health [46] and excess mortality [47] found in patients in Scotland with incentivised chronic diseases from more deprived communities may in part be explained by the lower uptake of evidence-based interventions, particularly in conditions associated with multi-morbidity and limitation of daily activity, for example, COPD. Other reasons for consistently high rates of non-attendance among indicators in patients with COPD or diabetes are not currently known. Some possible reasons may include patient unwillingness to attend appointments due to the number of repeated requests for attendance, or the duration of individual appointments. Transport difficulties, such as the cost of bus fares, or an inability to secure paid time off work to attend could also be contributory. Overall exceptions appeared higher in COPD, diabetes and stroke, which may be explained in part by the contribution made by higher ID rates (COPD and diabetes). Overall exceptions are calculated by the addition of the four categories described in Box 1. Therefore, high levels of 'Unsuitable', 'Registration date and diagnosis date' and 'Other' may account for higher overall exceptions.

Together, these data provide additional evidence [48, 49] that the inverse care law continues to operate in Scotland, despite over 10 years of the incentivised UK CDM contract designed to address health disparities.

### Implications for clinicians

Experience from other pay-for-performance systems suggests that patients from some minority groups have difficulty attending appointments and re-appointments due to problems with transport [50, 51, 53], poor understanding of recommendations [54] or costs [55]. Our findings underscore that tailored engagement approaches (e.g. phone calls or text reminders, or through house visits for housebound patients) may be merited for patients who are less likely to attend the practice [52]. Tailoring invitations, for example by offering flexible appointments or using methods other than re-sending invitation letters three times as stipulated in the CDM contract, is known to increase the uptake of general practice-based preventative care in ‘hard to reach’ groups in more deprived areas [52–56]. While individualised forms of engagement may require additional administrative time, effort and funding [45], practices are free to adopt new methods of engagement because the CDM contract does not constrain practices’ engagement approaches. More research is needed to identify methods to optimise engagement in deprived areas, given the growing evidence of the unintended consequence of severely ill patients being excluded from care [33, 57, 58].

### Implications for policymakers

UK primary care is seen as having a key role in improving clinical outcomes, particularly in deprived areas [16]. The incentivised CDM programme was seen as one mechanism for decreasing variation in the standards of healthcare and for potentially lessening inequalities in care provision [15, 16, 59, 60]. Our findings suggest there are socioeconomic inequalities in the uptake of best practice in clinical care for CDM and that the inverse equity hypothesis does not hold, contrary to previous findings [11, 34, 61, 62]. This reinforces calls to revise pay-for-performance programmes for CDM in a way that directly rewards reductions in inequalities [34, 63, 64], for example, by incentivising practices to reach patients who have more to gain but are less likely to attend their practice [76]. A revision of incentives based on need [47] or appointment systems, and increasing the skill mix in general practice to share the responsibility of managing chronic diseases [65], for example, by introducing practice-based, independent prescribing pharmacists who could visit patients in their homes, may help address the increased workload contributing to the underlying shortage of GPs.

As a result of the higher uptake of preventative healthcare in more affluent populations, international health improvement policies have recommended the use of targeted, preventative primary care [66–68], for example, using outreach workers to engage ‘hard to reach’ subgroups [69, 70]. Given the likely variation in invitation methods and associated successes or failures, contract revisions could include encouragement or incentivisation for practices to assess the acceptability and effectiveness of different engagement methods and use the method most suited to their patients. A comparison between UK and Californian programmes suggested that increasing financial rewards did not address health inequities: a revision of design and implementation was seen as more appropriate [18].

### Strengths and weaknesses of the study

We present findings from the majority of Scottish practices and describe, for the first time, practice-level time trends and factors predicting non-attendance due to ID among patients eligible to receive the UK’s incentivised CDM programme in Scottish primary care. The recording of non-attendance and refusal as ID for all 29 indicators over three recent contract years enabled us to evaluate these factors independent of changes to indicator definitions and to avoid selection bias. We accounted for changes to thresholds in our analysis, because of the finding that threshold changes influence exception rates [71].

The study has several weaknesses. Reporting systems are unable to differentiate ID non-attendance due to expressed refusal or when no reason is given. This limited our ability to make inferences about the extent to which patients chose not to attend rather than lacked the capacity or capability to attend, or did not receive an invitation, for example due to having moved house. Absolute rates of exceptions may be conditional on the indicators included because exception rates vary by indicator. However, this would not be expected to affect observed differences over time with the same indicators. In addition, the UK CDM contract rules only record the first exception reporting reason encountered in the information returned by practices, which may underestimate the true rate of ID. Participation in the pay-for-performance contract is voluntary, and there are differences in, for example, practice size and rurality, between practices participating and not participating in the UK pay-for-performance contract [77, 78]. Therefore, our conclusions, as with other practice-level analyses (Appendix 2) based on 83% of participating practices, may be limited to those participating in the UK pay-for-performance contract.

Our conclusions may not apply to other settings, such as those with market-based healthcare systems or low-income countries, and we did not consider dimensions of inequality such as ethnicity or gender, due to a lack of patient-level data.

### Comparison with existing literature

Median rates of ID and overall practice-level exception reporting were higher than those found in previous studies using data from English practices (Appendix 2). However, our ID results are not directly comparable because CDM programme reporting systems in England do not routinely differentiate ID from other exception reporting reasons, and include cases of ‘non-attendance after three invitations’ as ‘clinical – unsuitable’. Previous studies have not examined the same indicators over time, have included data from only one contract year, have not considered population achievement or have only examined data prior to 2010 [11, 17, 23, 33, 36], thereby limiting understanding of the inverse equity hypothesis in recent years of the UK CDM system.

Prior to the 2005 longitudinal study by Campbell *et al.* [72] and following the introduction of the incentivised CDM programme [11], analysis of practice-level data showed that the socioeconomic gradient in care quality narrowed, as anticipated by the inverse equity hypothesis [37]. However, studies used practice-level reported achievement without considering time trends in ID, overall exceptions or population achievement [10, 11, 38, 72]. Therefore, previous results may have masked underlying inequities [34].

Our practice-level findings are supported by patient-level data from other studies, which have suggested worsening of health disparities under the CDM contract through the exclusion of already disadvantaged groups, such as ethnic minorities with diabetes [73], patients with co-morbidities [32], and those at higher risk of diabetes complications [26, 74].

By using longitudinal data containing practice-level ID and overall exceptions together with population achievement, our study addresses calls from a recent systematic review [34] and adds further weight to emerging evidence that the incentivised system for CDM in the UK has not diminished inequalities [33, 34, 75]. More research is needed to explore the typology and engagement patterns of patients who are serially not engaging in incentivised CDM programmes so that approaches to engagement can be individualised.

## Conclusions

Overall and ID exception reporting rates have increased in Scotland. Rates of exception reporting including ID are higher in practices receiving higher remuneration, which may reflect practices being more adept at detecting and recording exceptions to avoid missing out on payments. Overall and ID exception reporting is higher in practices with greater numbers of patients from socioeconomically deprived areas. If, as would seem likely, ID rates represent non-engagement with CDM appointments, this would imply that ‘hard to reach’ patients are receiving suboptimal care. These results suggest there are widening socioeconomic and other inequalities and confound the inverse equity hypothesis. The CDM scheme is unlikely to be an example of a truly equitable public health intervention, suggesting a need for policymakers who are currently revising the scheme, or others contemplating such schemes in other countries, to consider including incentives for engaging with the 'hard to reach' who are most at risk of adverse outcomes if reducing health inequalities is deemed important.
